# A Rare Case of Giant Cell Tumour of Bilateral Achilles Tendon Sheath - Reconstruction with Dual Tendon Transfer: A Case Report

**DOI:** 10.5704/MOJ.2003.014

**Published:** 2020-03

**Authors:** P Samal, NC Mohapatra, J Mishra, A Mylarappa, P Das

**Affiliations:** 1 Department of Orthopaedic Surgery, Siksha O Anusandhan University Institute of Medical Sciences and SUM Hospital, Bhubaneswar, India; 2 Department of Orthopaedic, Sriram Chandra Bhanja Medical College and Hospital, Cuttack, India; 3 Department of Pathology, Siksha O Anusandhan University Institute of Medical Sciences and SUM Hospital, Bhubaneswar, India

**Keywords:** giant cell tumour, achilles tendon, reconstruction, bilateral

## Abstract

Giant cell tumour of tendon sheath is a benign soft tissue lesion most commonly found in the flexor aspect of hand and wrist. However, it is uncommon in foot and ankle and rare in bilateral achilles tendon. We report a case of 17-year-old female who presented with progressive enlargement of bilateral achilles tendon for six months. MRI findings showed that most of the tumour had intermediate to low signal intensity. Histopathology confirmed the diagnosis of giant cell tumour of tendon sheath. To help the patient regain the strength of the achilles tendon and walking abilities, a large area of tendon tumour was excised, followed by reconstruction with transfer of the peroneus brevis (PB) and posterior tibial (PT) tendon autograft. At two years follow-up, functional result was satisfactory.

## Introduction

Giant cell tumour of the tendon sheath (GCTTS) is seen commonly in the hand but their occurrence in the foot, ankle, knee or hip is less common^1^. Among the foot and ankle region, GCTTS of achilles tendon (AT) is less commonly reported and its bilateralism is rare. This case report highlights the rare occurrence of GCTTS and discussed a novel approach to reconstruct a large defect of the AT. We performed surgery on bilateral AT and the tumour affecting almost the entire length of tendon was widely resected. Both the peroneus brevis (PB) and posterior tibial (PT) tendon were harvested and transferred for reconstruction of AT. This surgical procedure was unique and provided reconstruction of large area defect with satisfactory functional result.

## Case Report

**Fig. 1:**
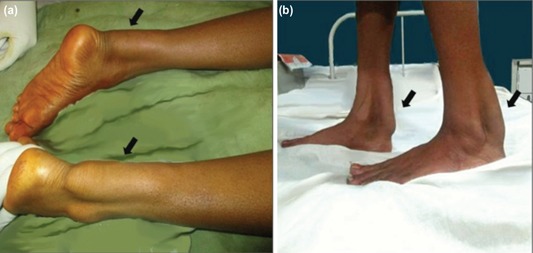
(a, b) Clinical photograph showing masses noted bilaterally on the posterior aspect of the legs above the calcaneus within the achilles tendon (AT) (arrow).

A 17-year-old female presented with a slow growing painless swelling on the posterior aspect of ankle for last six months. The swelling had progressively increased in the last two months with difficulty in walking. On examination, nontender, symmetrical, firm masses noted bilaterally above the calcaneus along the course of AT (Fig. 1A, B). A neurological evaluation was normal. Routine blood investigation results were normal. Magnetic resonance imaging (MRI) analysis of both ankles revealed bilateral enlarged AT showing intermediate to hypointensity on T1-weighted and T2- weighted images. Fine needle aspiration cytology smears showed giant cell rich lesion and histiocytes in singles and clumps scattered all over the fields.

**Fig. 2:**
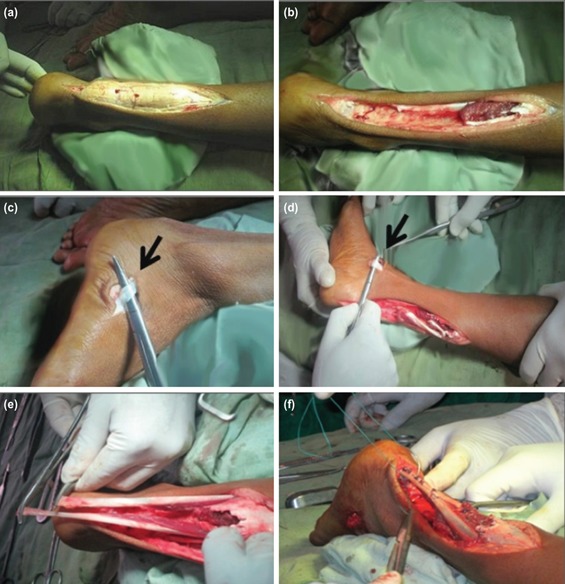
(a) Intra-operative photograph showing incision over the posterior aspect of the right AT. Note that the tendon was enlarged as a yellow-orange-red firm swelling. (b) Abnormal AT tendons were excised almost completely creating a large gap. (c) Incision over the base of fifth metatarsal enabling identification of the distal end of the peroneus brevis tendon. (d) The posterior tibial tendon was identified in the medial side and under the medial malleolus. (e) The peroneus brevis and posterior tibial tendon being delivered into the wound and approximated. (f) Both tendon ends after sutured with nonabsorbable, braided sutures and were fixed after tunnelled through a drill hole through the dorsal aspect of calcaneum.

**Fig. 3:**
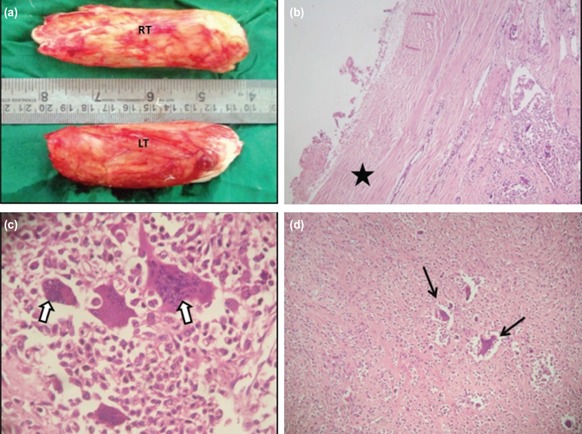
(a) Macroscopic photograph showing both right (11.5×3×2.5cm) and left (9.5×3.5× 2.5cm) side tumour mass with well circumscribed, smooth encapsulated, yellow-orange-red colour (b) Microscopic low power (haematoxylin and eosin, x5) view of GCTTS reveals well encapsulated tumour with thin fibrous capsule around it. (c) High-power (haematoxylin and eosin, x50) view of GCTTS demonstrating scattered groups of mononuclear cells with occasional osteoclastic multinucleated giant cells (white arrows), accumulation of histiocytes and hemosiderin laden macrophages are also seen. (d) Low-power view (haematoxylin and eosin, x10) of GCTTS reveals a prominent number of mononuclear cells with scattered multinucleated giant cells (arrows).

Operative procedure: (1) Incision: with the patient in prone position, a posteromedial skin incision of approximately 10cm was made till the insertion of AT. The exposed AT revealed large yellowish white nodular mass involving the entire distal part of both AT (Fig. 2A). (2) Excision of tumour: abnormal AT tendons were excised completely (Fig.2B). Gross specimen showed a large cylindrical yellowish orange-red soft tissue mass. The specimen measured 11.5×3×2.5cm on the right side and 9.5×3.5×2.5cm on the left side in the craniocaudal, transverse and anteroposterior dimensions (Fig. 3 A). After through excision, large defects (12.5cm right and 11cm left side) were created which were difficult to reconstruct by primary repair. (3) Tendon transfer: a 2.5cm longitudinal incision was made over the base of the fifth metatarsal (Fig. 2C). The PB tendon was identified, and then detached from its insertion and mobilised proximally. The tendon was then delivered through the posteromedial wound using gentle traction through the inferior peroneal retinaculum. The PT tendon was identified in the medial side and located under the medial malleolus through small incision (Fig. 2D). It was resected from the navicular bone and mobilised proximally. Both tendon ends sutured with nonabsorbable, braided sutures and fixed after tunneled through a drill hole through the dorsal aspect of calcaneus (Fig. 2 E, F). During fixation of graft, the foot was kept in equinus to reduce the gap and maintain AT in adequate tension. The closure was done in layers. The procedure was repeated in both the limbs in the same sitting.

The excised specimen sent for histopathological examination showed scattered groups of mononuclear cells with occasional osteoclastic multinucleated giant cells, foamy cells, accumulation of histiocytes and hemosiderin laden macrophages without mitotic figures (Fig 3B, C, D). Postoperatively, both the ankles were immobilised with a long leg cast in 20° of plantar flexion for the first six weeks. After removal of the cast, the patient was started with active physiotherapy. After three months, patient was allowed to bear weight progressively with the help of crutches within the limits of comfort. At six months follow-up the patient was able to walk without crutches. At one year follow-up, the patient was independently mobile. At two years follow-up, the functional outcome was satisfactory with an American Orthopaedic Foot and Ankle Society (AOFAS) ankle hind foot score of 92/100 as compared to Post-operatively, score of 76/100. The patient was able to walk on tiptoe with heel floor distance 5.5 and 5.2cm in right and left ankle, respectively. The range of motion (ROM) of both the ankle joints was 25° of dorsiflexion and 33° of plantar flexion.

## Discussion

GCTTS of the foot and ankle can occur in any age. The lesions are common in the forefoot, especially in the great toe. Because bilateral GCTTS of AT is rare, it may be misdiagnosed as other foot and ankle tumours. Approximately one third of all benign lesions are ganglions^1^. Complete surgical excision of affected tissue remains the mainstay of treatment. The gross pathological features include a well circumscribed lobulated or multi-nodular encapsulated mass with varying degrees of hyalinisation. On microscopy, the cellular infiltrate is constituted by macrophage-like mononuclear cells, epithelioid histiocyte-like cells, osteoclast-like giant cells. Because recurrence is thought to be due to incomplete excision of the tumour or satellite lesions, a balance must be maintained between salvaging tissue and adequate excision of tumour margins^2^. The defect in the achilles tendon can be filled either by cadaveric allograft or autograft^3^. Autograft is preferred in developing countries due to the limitation of allograft availability and pre-operative preparation. Among autogenous tendon transfer, methods such as PB transfer and flexor hallucis longus transfer are commonly used^4^.

For regaining better strength of the AT and functional abilities, we decided to undertake reconstruction using both PB and PT autograft transfer. Advantages include improved length of the tendon and strength of the muscle in comparison to that of other transfer candidates, especially in treatment of the excision and reconstruction of large tumours of the AT^5^. At final follow-up, the functional improvement was satisfactory in term of AOFAS score with ROM and able to walk tiptoe with good cosmetic appearance.

B/L GCTTS of AT is extremely rare. Other differential diagnosis should be excluded in the presence of bilateral enlargement of Achilles tendon. Reconstruction of large defects following wide excision of AT is often challenging in presence of limited graft sources. Combined transfer of both PB and PT tendon is a unique method to bridge the large defects with satisfactory result.
